# Emotionally challenging learning situations: medical students' experiences of autopsies

**DOI:** 10.5116/ijme.4f75.fb65

**Published:** 2012-03-31

**Authors:** Maria Weurlander, Max Scheja, Håkan Hult, Annika Wernerson

**Affiliations:** 1 Department of Learning, Informatics, Management and Ethics, Centre for Medical Education, Karolinska Institutet,; Stockholm, Sweden; 2 Department of Education, Stockholm University, Stockholm, Sweden; 3 Department of Clinical Science, Intervention and Technology (CLINTEC), Division of Renal Medicine, Karolinska Institutet, Stockholm, Sweden

**Keywords:** Student learning, medical education, emotional aspects, autopsy, qualitative analysis

## Abstract

**Objectives:**

To explore medical students’ experiences of an emotionally challenging learning situation: the autopsy.

**Methods:**

Qualitative data were collected by means of written accounts from seventeen students after their first and third autopsies and a group interview with seven students after their first autopsy. Data was interpreted using inductive thematic analysis.

**Results:**

Students experienced the autopsy in three ways: as an unnatural situation, as a practical exercise, and as a way to learn how pathologists work. Most students found the situation unpleasant, but some were overwhelmed. Their experiences were characterised by strong unpleasant emotions and closeness to the situation. The body was perceived as a human being, recently alive. Students who experienced the autopsy as a practical exercise saw it mainly as a part of the course and their goal was to learn anatomy and pathology. They seemed to objectify the body and distanced themselves from the situation. Students who approached the autopsy as a way to learn how pathologists work concentrated on professional aspects of the autopsy. The body was perceived as a patient rather than as a biological specimen.

**Conclusions:**

Autopsies are emotionally challenging learning situations. If students attend autopsies, they need to participate in several autopsies in order to learn about procedures and manifestations of pathological changes. Students need opportunities to discuss their experiences afterwards, and teachers need to be aware of how different students perceive the autopsies, and guide students through the procedure. Our findings emphasize the importance of investigating emotional aspects of medical education.

## Introduction

Autopsies have traditionally been important learning situations in medical education. Although autopsies have been used in medical education for a long time, studies investigating students’ attitudes and experiences of autopsies are scarce.^1^^-^^5^ Research on student learning in higher education tends to focus on cognitive aspects of learning, for instance, investigating experiences of understanding,^6^ approaches to learning,^7^ conceptual change8 or knowledge encapsulation.^9^ However, several researchers argue that motivation and emotion are equally important aspects of learning.^10^^,^^11^ During their undergraduate education, medical and health care students meet patients with serious diseases, and experience the suffering and death of some of these patients. Such encounters make strong impressions and can be very unpleasant experiences that students need to process in order to learn what is intended by the teachers.^12^^-^^14^ Artino and Durning^15^ argue that *“If we medical education researchers really want to improve medical education, we must broaden “what counts” as important and begin seriously exploring the role of emotion in learning.”* The scope of this study is to explore medical students’ experiences of an emotionally challenging learning situation: the autopsy.The first time students experience a dead human body in their undergraduate education is often during anatomy dissection. Studies on students’ reactions to anatomy dissections have revealed that many students found anatomy dissection disturbing, and described feelings of anxiety and disgust, with some suffering from nightmares and insomnia.^16^^-^^18^ Some students even had reactions resembling post-traumatic stress disorder.^16^ Moreover, the students said that they often had to deal with the unpleasant emotions alone, without support from teachers, and that they were concerned with how to balance the objectivity needed in the professional role with compassion for their patients.^17^^,^^18^ The feelings of unpleasantness and discomfort made learning difficult and students reported experiencing learning impairments.^16^Although autopsies have been a common learning activity in medical education, their prevalence has declined during the last decades.^19^^,^^20^ Clinical teachers and pathologists consider autopsies important learning opportunities for several reasons; to help students consolidate knowledge of anatomy and to understand mechanisms of diseases, but also to learn how a dead body is taken care of, to get an insight into how an autopsy is conducted, and to understand why autopsies are important when learning about diseases and treatments.^19^^,^^21^ However, studies investigating students’ experiences of autopsies are rare. Tazelaar et al found that students considered autopsies relevant for clinical practice because they learned to relate clinical and pathological findings and to view the process of disease.^1^ Benbow found that medical students considered the autopsy a useful and necessary teaching method in medical education.^2^^,^^3^ However, many students found the autopsy an unpleasant experience and some were genuinely distressed. These studies suggest that autopsies are emotionally challenging and demanding for students but offer no deeper insights into their learning and experiences of the situation. Recently, McNamee et al explored students’ experiences of autopsies during forensic medicine.^5^ They found that students expressed feelings of anxiety and discomfort, but also considered autopsies beneficial for learning anatomy, distinguishing between non-natural and natural causes of death, and improving knowledge of the autopsy procedure.

### Context of the study

This study was conducted in an undergraduate medical curriculum at a Swedish medical university. The curriculum, at the time of data collection, focused on basic sciences (e.g. cell biology, anatomy, physiology) in the first two years, followed by three and a half years of clinical courses (e.g. surgery, medicine). The pathology course, taught at the end of the second year, linked basic and clinical science, and several teaching and learning activities were used, e.g. lectures, formative assessments,^22^ case seminars^23^ and seminars during which students discussed microscopic images of tissue. During this course, the students also participated in autopsies. We became interested in emotional aspects of learning and therefore further explored students’ experiences of autopsies.During the nine-week pathology course, students participated in a minimum of three autopsies. The autopsies were conducted as a routine medical procedure by pathologists at the affiliated hospitals and the students attended the autopsies in groups of ^6^^-^^8^. The participants had all seen dead bodies before they attended autopsies since they had dissected cadavers in the anatomy lab during the anatomy course the previous year. To prepare students for their first autopsy, a teacher explained the purpose and the procedure of autopsies in a lecture, allowing students to ask questions and voice any concerns they might have. Apart from this, no further introduction to the autopsy room or the procedure was provided. The students did not actively participate in the autopsy procedure, but observed and touched the organs when allowed to.Since the autopsy is an under-researched learning situation that has a strong impact on students, especially emotionally, we wanted to explore and contribute to a better and more nuanced understanding of students’ experiences of this situation. The research questions addressed were: How do student experience autopsies? In what ways can autopsies offer important learning opportunities?

## Methods

### Design

In order to achieve the aim of the study, a qualitative research approach was chosen^24^^,^^26^ and an inductive thematic analysis was used to interpret data.^27^ The research was conducted within a constructivist research tradition, where data is viewed as being constructed in interaction between the researcher and informants, and the analysis is regarded as a process inevitably informed by the researcher’s prior knowledge and experiences.^28^^,^^29^ The constructivist research methodology is suitable when the aim of the research is to understand the meaning-making of individuals, and their perceptions or experiences of a certain situation or phenomenon.^26^

### Participants

The study reported here is part of a larger research project with the overall aim of investigating the interplay between teaching and learning in everyday teaching situations by studying students’ and teachers’ experiences of learning and understanding during the pathology course in a medical program. Medical students who were taking the pathology course during the autumn of 2007 were asked to participate in the study. Seventeen students from 70 volunteered to reflect on their experiences in writing, and another group of seven students participated in a group interview. The two groups of students varied in terms of gender (nine men and fifteen women), ethnic background and age (19-38 years), in order to ensure variation in their experiences of autopsies. Of the seventeen students who wrote down their reflections, four were international exchange students taking this particular course at this university. Although a convenience sample was used, the participants were representative of the study population as a whole in terms of gender, ethnical background and age.

### Data collection

Data were collected from the two groups of students described above, at five separate times during the course; early, in the middle, and at the end of the course. One of the authors (MW) followed the course and participated in many of the teaching sessions, and was the researcher that gathered the data. Parts of the data have previously been used.^22^ Data used for the present study was collected in the middle of the course, when the students had participated in autopsies. The written accounts were collected from seventeen students after they had attended their first, and then their third or fourth autopsies (two written accounts each, 34 in total). The additional group interview with seven students was conducted a few days after they had attended their first autopsy. The interview was held directly after a lecture. A relaxed atmosphere was sought and all students were encouraged to contribute. The purpose of combining the individual written accounts and the group interview was to ensure richer data. Group interviews have the advantage of stimulating interaction between participants.^26^The participants were asked to broadly describe their own experiences, in writing or during the interview, of the autopsies they had participated in. We asked open questions in order to stimulate reflection both in writing and during the interview. Examples of questions asked were: ‘Tell me about the autopsy’, ‘What are your thoughts about the autopsies you participated in?’ and ‘What did you learn by participating in the autopsies?’ To understand the teaching and learning context better, and to promote a relaxed atmosphere between researcher and participants, the first author followed the students during the course and participated as an observer in many of the teaching sessions, but was not involved in teaching or course management. The study followed national and international ethical guidelines on research involving human subjects (ethical approval 2007/1334-31/5 obtained from the local board of ethics), and written informed consent was obtained from all students prior to the data collection. The group interview was recorded electronically and fully transcribed.

### Analysis

All data, interview transcripts and written accounts, were analysed as one data set, using an inductive, thematic approach.^27^ Transcripts and written accounts were read several times in order for the researchers to familiarise themselves with the material. The analysis focused on the interpretational level, which meant that the researchers went beyond the manifest content that was explicitly said and written, and interpreted the latent content.^30^ The analysis was inspired by the hermeneutic circle and during the interpretation of the data, meaning units (sentences or short paragraphs) were related to the whole data set.^26^ The analysis was documented in notes and memos and made transparent for all researchers, and the themes were discussed and agreed upon by all to ensure that they were internally coherent and consistent.^27^ The trustworthiness of the analysis was enhanced by triangulation of data collection (on two separate occasions and using two ways of collecting data each time) and investigator triangulation (with different professional backgrounds such as pathologist, educational developer and educational researcher).^31^ Furthermore, a constant comparison between the themes and the original data was made in order to ensure a good fit between data and findings. The themes presented below represent ways of experiencing autopsies and comprise the empirical findings of this study.

### Results

Our findings show that all students found the autopsies important, but experienced them in different ways. We found three themes that represent different ways of experiencing autopsies: as an unnatural situation, as a practical exercise, and as a way to see how pathologists work. These three themes differed in terms of what aspects of the experience were in focus, how the body was perceived, and to what degree students distanced themselves from the situation (Table 1). It also seemed that students became more accustomed to the situation after they had participated in several autopsies. The different themes, or ways of experiencing autopsies, are described in more detail below and illustrated by quotes.

**Table 1:** An overview of the themes representing different ways of experiencing the autopsy

Theme	Focus	Perceptions of the body	Emotional reaction	Learning	Dimension of closeness - distance
Autopsy as an unnatural situation	The unnaturalness of the situation	A human being – with family and friends	Strong emotional experience - unpleasantness	Some knowledge of anatomy and pathology, and how autopsies are performed	Emotionally close – physically distant
Autopsy as a practical exercise	The pathology course	A specimen – objectified	Emotions of unpleasantness, but able to cope – interested	Repetition of anatomy and learning pathology, clinical relevance	Emotionally distant – physically close
Autopsy as a way to see howpathologists work	The clinical practice	A patient	Emotions of unpleasantness, but able to cope – interested	Mainly how autopsies are performed and cause of death is determined	Emotionally distant – physically close

### Autopsies as an unnatural situation

For some students, the first autopsy was such an emotionally strong experience that they had difficulty coping with their anxiety. Thus, the focus of their experience was the unnaturalness and awkwardness of the situation. Students felt their own body react strongly to the situation, for example experiencing feelings of nausea and fear of fainting. These students found it hard to concentrate on what was going on during the autopsy and some students felt that they did not learn much from the experience, while others learned some anatomy and pathology. The body was perceived as a human being that had recently led a normal life with family and friends, and the students reacted strongly to how this person’s body was dealt with during the autopsy procedure. They used words such as “bizarre” and “surreal” to describe their experiences. The teacher had an important role in guiding students through the procedure.

Clear guidance helped the students to have a meaningful experience despite the strong feelings of anxiety. However, some students experienced a lack of guidance, which made it even more difficult to cope with the unpleasantness of the situation. This theme is characterized by closeness to the situation. The dissected body, the smells, and the emotional reactions seemed to overwhelm the students.

*“I found the autopsy really awkward. I felt nauseous and had to keep moving in order not to faint. But I found it instructive, at least when it comes to anatomy.”* Student 1, written account

*“I was looking at a person that went to work yesterday or the day before that and they feel so alive, in some way, and then they just lie there, cut open.”* Student 2, group interview

*“And at this very moment, when we stand here (in the autopsy room) there are family and friends who are really sad and heartbroken”* Student 3, group interview

### Autopsies as a practical exercise

In this theme the focus of the experience was the autopsy as a course activity with the primary purpose of learning pathology and rehearsing anatomy. The opportunities to see, to smell and touch the organs were important for the students’ learning. The autopsy was found to give a clinical connection to the course content (pathology) which seemed to help students in their learning. Autopsies where students saw “new” pathological changes were considered more meaningful. The body was perceived as a biological specimen, and in this respect it was objectified. The students talked about the body using words such as “specimen”, “body” or only in terms of specific organs. The students found the autopsy emotionally challenging, but seemed to cope with the situation by distancing themselves mentally and emotionally and by focusing on learning about the studied organs and pathologies. A good introduction and clear guidance through the procedure by the teacher was important for the students; it helped them to focus on what was found and they could relate to previous autopsies. Another aspect that contributed to the students’ experience was the group size. A small group was preferred because that made it possible for all to come close to the table and touch and see the organs in detail. The quotes below illustrate this way of experiencing autopsies.

*“Awkward, maybe first of all to distinguish between post-mortem changes and pathological changes […] Secondly, it is good to get some anatomy revision by seeing the corpse and the organs. […] It was interesting to see with my own eyes the pathological changes that we had heard about, for instance different types of necroses. [...] The second autopsy was also very interesting. And by recalling the summary of our findings from the first autopsy right before we started with the second one I could compare the two autopsies and appreciate the differences. […] When it comes to the third autopsy, I think that it was a little bit boring. There wasn't much difference from the earlier ones and I don't think I learned a lot during those three hours in the autopsy room.”* Student 3, written account

*“Sure, it was a bit strange to see all the blood and the body in the unusual state, and it was difficult concentrating on other things apart from the bad smell. But if you focused on why you were there and what you actually saw, you could forgot the unpleasantness of the situation, from time to time, and could learn a little. It was a surprisingly good opportunity to rehearse anatomy.”* Student 4, written account

The quotes above illustrate how students managed to distance themselves from the unpleasantness of the situation, and found the autopsy an opportunity to engage in the study of anatomy, to learn pathology and to develop understanding of how diseases manifest themselves in the human body. For these students, the autopsies seemed to be a meaningful learning experience.

### Autopsies as a way to see how pathologists work

The focus in the third theme was on the autopsy as a professional activity, a part of the clinical work of pathologists, and the students appreciated the opportunity to participate and learn how the procedure was conducted. The purpose of the autopsy, to determine the cause of death, and the procedure around the autopsies, seemed to be the focus of students’ experience in this theme. Consequently, students learned mainly how autopsies are carried out, and what pathologists and technicians do respectively. The body was seen as a patient, and students used words such as “body”, “patient” and “person” when they described their experiences. The students found the autopsies interesting, and by focusing on the procedure and finding the cause of death, they coped with the unpleasantness of the situation. This theme was also characterized by students distancing themselves from the situation, trying not to involve their emotions. The importance of a good introduction and clear guidance from the teacher were also found in this theme.

*“You learned a lot in terms of which organs the pathologist looks at […] We went through the things that are important for the pathologist during the autopsy and the different procedures during the first autopsy, and also basic macro-pathology”* Student 5, written account

*“By the second and third autopsies I think that you realized that they used the same procedure in each autopsy, regardless of the patient’s history and how the patient died”* Student 6, written account

### Shifting focus

As illustrated above, attending the first autopsy seemed to have a strong emotional impact on many students and they needed to process their experiences in order to cope with the situation and learn what was intended by the teachers. The students got used to the situation, and the subsequent autopsies were not as difficult as the first one. During the later autopsies it seems that the students either related to the purpose of the autopsy, or focused on learning anatomy and pathology. In doing so, they shifted focus from the unnaturalness of the situation to perceiving the autopsy as a practical exercise (the second theme) or as a way to see how pathologists work (the third theme). The quotes below are typical of a student that at first found the autopsy unnatural and unpleasant. After attending three autopsies the student mentioned that he/she had become accustomed to the situation and had learned some pathology. This student has moved from experiencing the autopsy as an unnatural situation to seeing it as a practical exercise.

“An autopsy is an unnatural situation and is in many ways awkward. Even though we have dissected before, it is not the same. The specimens [in anatomy lab] are treated and in some ways as being less human. […] At the autopsy, the specimens are more human, they have smells and feel more alive”.

*“At the third autopsy […] we were only four. You could participate in the autopsy and it was a much better opportunity for learning. […] I don’t know if I learned anything special at the different autopsies. Maybe how necrotic tissue looks, how […] tissue looks after a heart attack etc.”* Student 7, written account

Another example of a shift in experiencing the autopsy is illustrated by the following quotes. This student also first experienced the autopsy as an unnatural situation but moved towards experiencing the autopsy as a way to see how pathologists work.

*“During the first autopsy I really had to deal with my feelings and get used to the entire procedure. It was also a strange feeling to be in the presence of a person who had recently died. […] The second time, I noticed it was much easier to be there and I could concentrate on what was really going on. I asked some questions and could really try to think with the pathologist and the rest of the group about the cause of death. […] It is so interesting to see an autopsy; it is a unique chance to the see the entire inside of a human body and to see all kinds of defects.”* Student 8, written accounts

## Discussion

The purpose of our study was to explore medical students’ experiences of autopsies as an example of an emotionally challenging learning situation. Our findings show that the students found autopsies to be an important part of medical education, but experienced them differently. Some students experienced the autopsy as an unnatural situation, and the focus was on their own feelings of anxiety and surrealism and their physical reactions such as nausea. These students found the whole situation overwhelming and did not seem to learn much. Some students, on the other hand, experienced the autopsy mainly as a practical exercise, part of the pathology course they were taking, and they learned about anatomy and pathological changes. Other students had the professional aspects of the autopsy in focus and experienced it as a way to see the work of pathologists. These students mainly learned about the autopsy procedure and how pathologists look for the cause of death. For the majority of the students the autopsy was an emotionally challenging learning situation, but for some it was indeed difficult to cope with the emotional aspects. The strong emotions evoked in the autopsy room must be dealt with in order to engage in productive learning. Emotion, cognition and motivation interact in complex ways and strong negative emotions, such as anxiety, have been found to make learning more difficult.^32^^,^^33^Our findings suggest that students got used to the situation in the autopsy room and learned to cope with the associated negative emotions, and this was also reported by McNamee et al.^5^ Students who experienced the autopsy as an unnatural situation seemed to shift focus after they had participated in several autopsies. They shifted either to perceiving the autopsy as a practical exercise or as a way to see how pathologists work. There does not seem to be a linear development with phases where students first experience the autopsy as an unnatural situation, then as a practical exercise, and finally as part of the clinical work of pathologists. Instead, it is more likely that students’ experiences of autopsies depend on what aspects they focus on (for example learning course content or observing clinical practice). Thus, some aspects become part of the foreground of their experience, and other aspects remain in the background. Students seem to contextualise the same situation differently and the aspects they pay attention to influence their interpretation of the situation and what they gain from the experience. This correlates to other findings where students interpreted the same task in different ways ending up working on different problems.34 The themes described are therefore different in terms of what aspects of the autopsy students pay attention to, and that these aspects can change over time, but we do not see the themes as hierarchical or representing different developmental phases that students go through. McNamee et al found that students went “through a degree of ‘desensitisation’, which had set in by the second autopsy” and they were then able to concentrate on other aspects of the autopsy.^5^ This is similar to our findings that students shift focus after attending several autopsies.The dimensions of closeness and distance are present in all three themes, but in different ways. When students found the autopsy an unpleasant and an unnatural situation they seemed to become very involved in the situation mentally and emotionally. The body was perceived as a human being and a person that had recently lived. Some students reacted to this by taking a step back from the autopsy table and the dissected body lying there, thus distancing themselves physically. Students who experienced the autopsy as a practical exercise seemed to prefer to be physically close to the autopsy table and to focus on specific organs and at the same time keep an emotional distance by objectifying the body. The same pattern of being emotionally distant but physically close was found with students who experienced the autopsy as a way to see how pathologists work. The students in our study seemed to struggle to become detached and manage their negative emotions, and at the same time allow themselves to approach the body. The dimensions of closeness and distance have also been reported by Smith & Kleinman.^12^ They studied how medical students managed the physical intimacy and contact with the human body during contact with both living and dead patients, and how students dealt with their emotional reactions. Smith & Kleinman found that students experienced a range of negative feelings and students managed their emotions by either focusing on specific body parts or organs rather than the person (coming close), or by objectifying the body (distancing).The objectification of the body as a way of coping has been described previously, and our findings correspond to earlier research showing that the affective socialization of students and the enculturation into medical practice starts early in medical education.^4^^,^^12^^,^^35^^,^^36^ The anatomy dissections and autopsies have a strong impact on students and are part of students’ initiation into the practice of medicine. They learn to cope with disturbing and unpleasant emotions and start to develop a detached concern.^4^^,^^5^^,^^35^ However, coping with these emotions seems to be left to students, without deliberate support from teachers, and students may have a fear of becoming desensitized, which could influence their empathy and compassion for their future patients.^18^ In the present study, no attempts were made by the course leader to support students emotionally by offering them opportunities during scheduled class time to discuss their experiences with peers and teachers. The finding that students have to deal with their emotions alone has also been reported in other studies.^12^^,^^17^^,^^18^The autopsy represents an emotionally challenging learning situation, but medical and health care students encounter many situations during their training that might evoke strong feelings. They need to be able to cope with their emotions without negatively affecting the empathy for their patients. It is therefore worrying that students have to deal with such experiences alone. One possible reason is that teachers do not themselves feel entirely comfortable in supporting students and discussing emotional aspects of autopsies and the death of patients.Autopsies can be important learning opportunities where students learn about anatomy, pathology, relate clinical and pathological findings, and how the autopsy is conducted.^1^^-^^3^^,^^5^ Our findings suggest that in order to learn from autopsies students need to shift focus, from the overwhelming unnaturalness of the situation to focusing on anatomy and pathology, or the autopsy procedure and how pathologists work. Also, students’ learning may be facilitated by better emotional preparation before their first autopsy and an opportunity to reflect on and discuss their experiences afterwards.^18^Teachers play a central role in all teaching and learning activities, and especially in emotionally challenging situations. A situation that evokes strong emotions is likely to be remembered for a very long time, either negatively or positively. In our study, some teachers helped students by guiding them through the autopsy procedures and clarifying the purpose. This seemed to help the students to cope constructively with the situation, which in turn created better opportunities for learning, for instance by increasing their interest. The autopsy can be a useful learning opportunity where students not only learn anatomy, pathology and the procedure, but where socialization into medical practice occurs in a meaningful way. Together teachers and students may discuss attitudes towards the patient and the human body in medical practice and how to deal with the different emotions that arise. Teachers are role models in how they approach the body in the autopsy room, and how they talk to and about patients will have an impact on students’ professional socialization.

### Limitations of the study

Although the number of participants was limited, which is a necessity in a qualitative inquiry, we argue that the study illustrates important aspects of students’ experiences during medical education and emotional aspects of learning. We have explored students’ experiences of an emotionally challenging learning situation in a certain context. The usefulness of our findings in other contexts does of course depend on the local situation and must be considered in relation to that particular context and situation.

### Implications for practice

Our findings raise important questions regarding medical and health care students’ experiences of emotionally challenging situations during their undergraduate education. Students found the autopsy emotionally difficult, and some had trouble coping with the situation, while others distanced themselves emotionally by objectifying the body. Autopsies probably are one of the most emotionally challenging learning situations during medical education, and our findings can help teachers and educators to understand how students may react to other emotionally challenging learning situations. The purpose of autopsy teaching in the present course was to help students to develop a deeper understanding of how pathological processes affect the human body, and to understand the mechanisms of different diseases and of death. Our findings indicate that many students do not reach this understanding during their first autopsy. The implications of our study are as follows:

1. If autopsies are a part of undergraduate training of medical students, they need to participate in several autopsies. It takes time to get used to the situation, cope emotionally and to learn from the experience
2. Students would benefit from better and more thoughtful emotional preparation before entering the autopsy room for the first time, and should have the opportunity to discuss and voice concerns with peers and teachers afterwards, e.g. in some form of small group seminar where students get help to make sense of their experience.
3. Teachers and pathologists need to be trained in how to best guide students through the autopsy, how they can help students to understand the purpose of autopsies and focus on learning about the human body as a system, and be aware of the different ways students can experience the autopsy.

Furthermore, medical and health care students encounter many situations during their undergraduate education that might evoke strong emotional reactions. Teachers and educators have important roles in guiding students and should discuss difficult emotional aspects of medical and health care practice, such as human dignity, death, grief and how to deal with emotions, both their own and those of patients.^37^ More research is needed in this area, so that we can prepare students in better ways for the emotional aspects of their future professional role.

## Conflict of Interest

The authors declare that they have no conflict of interest.
